# Carotid intima–media thickness and cardiovascular risk factors in healthy volunteers

**DOI:** 10.1186/s13089-021-00218-6

**Published:** 2021-03-11

**Authors:** Birgit-Christiane Zyriax, Kira Dransfeld, Eberhard Windler

**Affiliations:** 1 Midwifery Science—Health Care Research and Prevention, Preventive Medicine and Nutrition, German Center for Health Services Research in Dermatology (CVderm), University Medical Center Hamburg-Eppendorf (UKE), Hamburg, Germany; 2 Institute for Health Services Research in Dermatology and Nursing (IVDP), University Medical Center Hamburg-Eppendorf (UKE), Hamburg, Germany; 3 Midwifery Science—Health Care Research and Prevention, Preventive Medicine and Nutrition, Institute for Health Care Research in Dermatology and Nursing (IVDP), University Medical Center Hamburg-Eppendorf (UKE), Martinistr. 52—Bldg. N26, D-20246 Hamburg, Germany

**Keywords:** Carotid intima–media thickness (CIMT), Cardiovascular, Risk score, Atherosclerosis, Cardiovascular risk

## Abstract

**Background:**

Assessment of cardiovascular risk by scores lacks sensitivity and leaves the majority of future cardiovascular patients unidentified particularly individuals at low cardiovascular risk. The present analysis investigates into the correlation of carotid intima–media thickness (CIMT) and cardiovascular risk factors and derived scores as to the potential of improved cardiovascular risk prediction by combining the two.

**Methods:**

The Stress, Atherosclerosis and ECG Study (STRATEGY) is a cross-sectional study of selectively healthy 107 women and 106 men without diagnosed and treated cardiovascular risk factors evenly distributed between 30 and 70 years. CIMT was determined by evaluating B-mode ultrasonograms offline according to a standardized protocol. The unpaired *t*-test was used to compare normal-distributed continuous variables, the Chi-squared test for normal-distributed categorical variables and the Mann–Whitney *U* test for non-normal distributed continuous variables. The association between risk prediction scores and CIMT was calculated by the Spearman rank correlation coefficient. Pearson correlation coefficient was used for the correlation between cardiovascular risk factors and CIMT. A multiple linear regression analysis was executed for the association of cardiovascular risk factors and CIMT.

**Results:**

Age, systolic blood pressure, fasting glucose, total, LDL- and non-HDL-cholesterol and waist circumference were significantly associated with CIMT (each *P* ≤ 0.03). The Framingham Risk Score, the Prospective Cardiovascular Münster Study Score and the European Society of Cardiology Score correlated significantly but only moderately with CIMT. The Framingham Risk Score considering BMI correlated most strongly and predicted 27% of the CIMT variance in men and 20% in women.

**Conclusion:**

In individuals without overt cardiovascular risk factors and thus at low cardiovascular risk, CIMT and cardiovascular risk factors correlated only partially suggesting that combining CIMT and conventional risk factors or common derived scores may improve risk prediction in individuals at low cardiovascular risk. The clinical benefit as to cardiovascular events of such combined risk prediction needs to be explored in large prospective cohorts of still healthy low-risk volunteers. DRKS ID DRKS00015209 07/02/2019 retrospectively registered https://www.drks.de/drks_web/navigate.do?navigationId=resultsExt

## Introduction

The early identification of individuals at risk for future cardiovascular events is of vital importance since early correction of emerging risk factors is more efficient than therapy of advanced atherosclerotic vascular disease. Risk assessment by scores lacks sensitivity and leaves the majority of future cardiovascular patients unidentified particularly in individuals at low risk without overt risk factors [1–3].

Noninvasive measurement of structural changes in straight arterial segments as carotid intima–media thickness (CIMT) is a well-established surrogate marker of early stages of subclinical atherosclerosis. It helps to identify individuals at risk of future clinical endpoints including coronary and cerebrovascular events earlier than established risk factors [4–7]. However, current evidence does not satisfactorily support the routine measurement of subclinical atherosclerosis in primary prevention [8].

The 10-year cardiovascular risk perception of widely used risk scores is mainly based on the presence of classical risk factors as lipid values, hypertension, diabetes, smoking, age and gender [1–3]. In observational longitudinal studies, a clear relationship was observed between scores and the incidence of cardiovascular events [3].

However, the predictive value is limited, as the majority of coronary events occur in individuals of low- and intermediate-risk based on risk scores [2, 4]. Since the absolute risk is low in these risk categories, it would be beneficial to improve the specificity of prediction to allocate preventive measures more efficiently.

Thus, the aim of the present explorative analysis of data from the Stress, Atherosclerosis and ECG Study (STRATEGY) representing a population at low cardiovascular risk that has not yet developed overt cardiovascular risk factors was to analyze the concordance of CIMT and common risk prediction scores regarding risk perception as to the possible benefit of combining the two.

## Patients and methods

### Design and recruitment

Methods applied in STRATEGY have been described in detail [9]. STRATEGY is a cross-sectional study of individuals without overt cardiovascular risk factors, 107 (50.2%) women and 106 (49.8%) men aged 30–70 years recruited in Hamburg through a health insurance by a flyer randomly included in regular mailings between September 2006 and March 2007. The data previously analyzed and published for other issues were re-used because of the low use of statins in primary prevention at that time and the opportunity to make use of the validated procedure of determining CIMT of the Study of Health in Pomerania. Participants without acute or chronic diseases, or any medication except for hormone replacement therapy or oral contraceptives and thyroxin for stably substituted hypothyroidism were invited for a visit. Up to at least 25 participants per age decade and gender were consecutively included. The data of 2 out of 215 individuals were excluded from the analysis because of missing data.

### Data collection

#### Assessment of anthropometric and clinical data

All interviews and physical examinations were performed by the same trained investigator. Body height and weight were measured to the nearest 0.5 cm or 0.1 kg, respectively, body mass index (BMI) was calculated as weight [kg]/(height [m])^2^. 'Smokers' refers to current smokers and 'non-smokers' summarizes ex- and never-smokers. A fasting morning blood sample was taken, immediately centrifuged at 4 °C and stored at 4 °C in tubes containing 1 mg fluoride per ml blood. Fasting plasma glucose, high-density lipoprotein (HDL-) cholesterol, and triglycerides were determined by standard techniques. Low-density lipoprotein (LDL-) cholesterol was calculated using the Friedewald formula. Blood pressure was taken in a sitting position in the morning three times in 1-min intervals after at least 5 min of rest. The results of the second and third measurement were averaged and used for the present analysis. Hypertension was defined as blood pressure ≥ 140/ ≥ 90 mmHg [9].

#### Assessment of intima–media thickness (CIMT)

CIMT was assessed by the standardized method of the Study of Health in Pomerania (SHIP) as previously applied and reported [9–11]. IMT of the common carotid artery was recorded on both sides between the carotid bulb origin and a point 10 mm proximal using high resolution B-mode ultrasound (General Electric Vivid 3 Expert with a 7.5-MHz linear array transducer). The CIMT was assessed offline as the average of at least five consecutive measurements in 1-mm steps on both sides of the distance from the media–adventitia interface to the intima–lumen interface on the far wall in a region free of plaques—only one plaque in one participant was detected. The offline measurements were performed at the center of the Study of Health in Pomerania (SHIP), Greifswald, by a single reader, who was trained according to the standardized protocol of the study as reported in detail [9, 10]*.*

#### Risk assessment by cardiovascular risk prediction scores

For each participant two variants of three established scores assessing 10-year cardiovascular risk were calculated [12–14] (Table 1).

**Table 1:** Predictors of cardiovascular risk calculated according to the Framingham Risk Score including lipids or body mass index, the Prospective Cardiovascular Münster Study Score including lipids or body mass index, the German version of the European Society of Cardiology Score including total cholesterol or total cholesterol/high-density lipoprotein

Predictors	FRS		PROCAM		ESC	
	Lipid	BMI	Lipid	BMI	TC	TC/HDL
Age, gender, smoking, systolic blood pressure	X	X	X	X	X	X
Diabetes	X	X	X	X		
Antihypertensives	X	X		X		
Family history			X	X		
Cholesterol, triglycerides	Total, HDL		LDL, HDL, Triglycerides		Total	Total, HDL
Anthropometry		X		Height, weight		
Calculated 10-year risk	Cardiovascular diseases		Myocardial infarction		Cardiovascular death including fatal stroke	

*FRS* Framingham Risk Score, *PROCAM* Prospective Cardiovascular Münster Study Score, *ESC* European Society of Cardiology Score, *BMI* body mass index, *TC* total cholesterol, *HDL* high-density lipoprotein

### Statistical analyses

The explorative analysis was performed on the entire study population and for each gender. Comparisons of means between groups were performed using unpaired *t*-test for normal-distributed continuous variables, Chi-squared test for normal-distributed categorical variables and Mann–Whitney *U* test for non-normal distributed continuous variables. The association between common risk prediction scores and CIMT was calculated by the Spearman rank correlation coefficient. Pearson correlation coefficient was used for the correlation between cardiovascular risk factors and CIMT. A multiple linear regression analysis was executed for the association between cardiovascular risk factors and CIMT. *P*-values of 0.05 were used as the cut-off for statistical significance. All analyses were conducted using SPSS 24.

## Results

### Baseline characteristics

Table 2 gives the characteristics of the population of the STRATEGY study. Compared to men, women were characterized by lower LDL-cholesterol, triglycerides, fasting glucose and blood pressure and higher HDL-cholesterol. However, on average most laboratory values were within the range of normal.

**Table 2:** Characteristics of the study population

	Men	Women	Significance
	*n* = 106	*n* = 107	*P*-value
Age [years]	49.6 ± 11.3	49.4 ± 10.6	0.893
Smokers [%]	8.5	11.2	0.505
Systolic blood pressure [mmHg]	129.2 ± 13.9	123.2 ± 17.9	0.007
Total cholesterol [mg/dl]	210.5 ± 35.7	207.1 ± 35.3	0.485
LDL-cholesterol [mg/dl]	124.1 ± 31.0	110.4 ± 32.2	0.002
HDL-cholesterol [mg/dl]	63.2 ± 15.8	80.4 ± 17.6	< 0.001
Total cholesterol/HDL ratio	3.5 ± 1.03	2.7 ± 0.72	< 0.001
Triglycerides [mg/dl]	116.6 ± 66.6	85.0 ± 36.6	< 0.001
Fasting glucose [mg/dl]	92.3 ± 9.5	87.5 ± 7.8	< 0.001
BMI [kg/m^2^]	26.2 ± 3.2	23.9 ± 3.3	< 0.001
Height [cm]	180.6 ± 7.2	166.6 ± 6.8	< 0.001
Weight [kg]	85.6 ± 12.1	66.5 ± 10.0	< 0.001
Waist circumference [cm]	96.0 ± 10.6	81.6 ± 9.4	< 0.001
Carotid intima–media thickness [mm]	0.70 ± 0.1	0.68 ± 0.09	0.103

*LDL* low-density lipoprotein, *HDL* high-density lipoprotein, *BMI* body mass index

### CIMT and cardiovascular risk factors

Similarly in both gender, the average CIMT was slightly elevated compared to 0.3–0.5 mm of infants [15–17] (Table 2). Age, systolic blood pressure, fasting glucose, total, LDL- and non-HDL-cholesterol and waist circumference correlated significantly though weakly to moderately with CIMT in the study population (Table 3). When analyses were considered separately for men and women, some correlations lost their significance probably due to small sample size.

**Table 3:** Pearson correlation between single common cardiovascular risk factors and carotid intima–media thickness in men and women

Risk factor	Total (*n* = 213)	Men (*n* = 106)	Women (*n* = 107)
Age	0.510 (*P* < 0.001)	0.531 (*P* < 0.001)	0.489 (*P* < 0.001)
Smoking status	0.017 (*P* = 0.806)	− 0.050 (*P* = 0.610)	0.073 (*P* = 0.455)
Systolic blood pressure	0.318 (*P* < 0.001)	0.298 (*P* = 0.002)	0.319 (*P* = 0.001)
Total cholesterol	0.205 (*P* = 0.003)	0.279 (*P* = 0.004)	0.114 (*P* = 0.242)
LDL-cholesterol	0.154 (*P* = 0.024)	0.186 (*P* = 0.056)	0.080 (*P* = 0.412)
HDL-cholesterol	0.026 (*P* = 0.709)	0.126 (*P* = 0.197)	0.050 (*P* = 0.614)
TC/HDL-cholesterol	0.116 (*P* = 0.092)	0.098 (*P* = 0.315)	0.040 (*P* = 0.682)
Non-HDL-cholesterol	0.182 (*P* = 0.008)	0.217 (*P* = 0.026)	0.091 (*P* = 0.354)
Triglycerides	0.127 (*P* = 0.065)	0.167 (*P* = 0.089)	-0.030 (*P* = 0.757)
Height	− 0.014 (*P* = 0.841)	− 0.140 (*P* = 0.152)	− 0.124 (*P* = 0.202)
Weight	0.048 (*P* = 0.489)	− 0.053 (*P* = 0.592)	− 0.926 (*P* = 0.926)
BMI	0.075 (*P* = 0.274)	0.024 (*P* = 0.808)	0.060 (*P* = 0.539)
Waist circumference	0.148 (*P* = 0.031)	0.162 (*P* = 0.096)	0.028 (*P* = 0.774)
Fasting glucose	0.249 (*P* < 0.001)	0.287 (*P* = 0.003)	0.150 (*P* = 0.124)

*LDL* low-density lipoprotein, *HDL* high-density lipoprotein, *TC* total cholesterol, *BMI* body mass index

Based on univariate linear regression, age explained 28.2% of the variance of CIMT in men and 23.9% in women. Systolic blood pressure predicted 8.9% of the variance of CIMT in men and 10.2% in women. In the total population 7.8% of the variance in CIMT was explained by total cholesterol. Based on univariate linear regression, age, systolic blood pressure and total cholesterol were the strongest predictors of the variance of CIMT, in men 28.2, 8.9, and 7.8%, respectively, and in women 23.9, 10.2, and 1.3%, respectively. Each year of life translated into an additional 0.004 mm CIMT, each 1 mmHg systolic blood pressure into an additional 0.002 mm CIMT, and each additional 10 mg total cholesterol correlated with a 0.007 mm higher CIMT in men and 0.003 mm in women. In a multivariate regression model the increase of CIMT was moderately predicted by age and systolic blood pressure, whereas all other common cardiovascular risk factors missed significance (Table 4). Since age may mask the effect of risk factors that increase with age, the multivariate regression was conducted omitting age, which revealed a stronger association of some risk factors with CIMT, particularly systolic blood pressure.

**Table 4:** Multivariate regression model of cardiovascular risk factors and carotid intima–media thickness considering and omitting age

Model parameters	Multivariate model including age		Multivariate model excluding age	
	Standardized regression coefficient	*P*-value	Standardized regression coefficient	*P*-value
Age	0.467	< 0.001	**–**	**–**
Male gender	− 0.089	0.199	− 0.074	0.327
Smoking status	− 0.007	0.900	− 0.012	0.855
Systolic blood pressure	0.134	0.048	0.263	< 0.001
LDL-cholesterol	− 0.078	0.262	0.069	0.342
HDL-cholesterol	0.033	0.663	0.142	0.086
Triglycerides	0.102	0.162	0.078	0.332
BMI	− 0.041	0.550	− 0.072	0.337
Fasting glucose	0.040	0.566	0.157	0.033

*LDL* low-density lipoprotein, *HDL* high-density lipoprotein, *BMI* body mass index

### CIMT and cardiovascular risk prediction scores

Taking in account all major risk factors may have a stronger impact on the CIMT than considering each separately. Therefore, risk scores were calculated using the FRS, PROCAM or ESC and their respective variants (Table 1). The resultant scores were significantly higher for men than women (*P* < 0.001). Scores of FRS and PROCAM using weight and height (BMI) instead of lipids were significantly higher in both genders (*P* < 0.05). Still, both score variants and the ESC correlated strongly with each other (*P* < 0.001). Accordingly, all cardiovascular risk scores correlated significantly and positively with CIMT (Table 5). Compared to PROCAM the associations between ESC and the FRS and CIMT were slightly but not significantly stronger.

**Table 5:** Spearman rank correlation between cardiovascular risk scores and carotid intima–media thickness (*P* < 0.001 for each correlation)

Risk scores	Total	Men	Women
	*n* = 213	*n* = 106	*n* = 107
FRS-Lipids	0.513	0.564	0.444
FRS-BMI	0.530	0.589	0.466
PROCAM-Lipids	0.464	0.509	0.437
PROCAM-BMI	0.479	0.543	0.447
ESC-TC/HDL	0.527	0.547	0.462
ESC-TC	0.507	0.561	0.410

*FRS* Framingham Risk Score, *PROCAM* Prospective Cardiovascular Münster Study Score, *ESC* European Society of Cardiology Score, *BMI* body mass index, *TC* total cholesterol

**Fig. 1:**
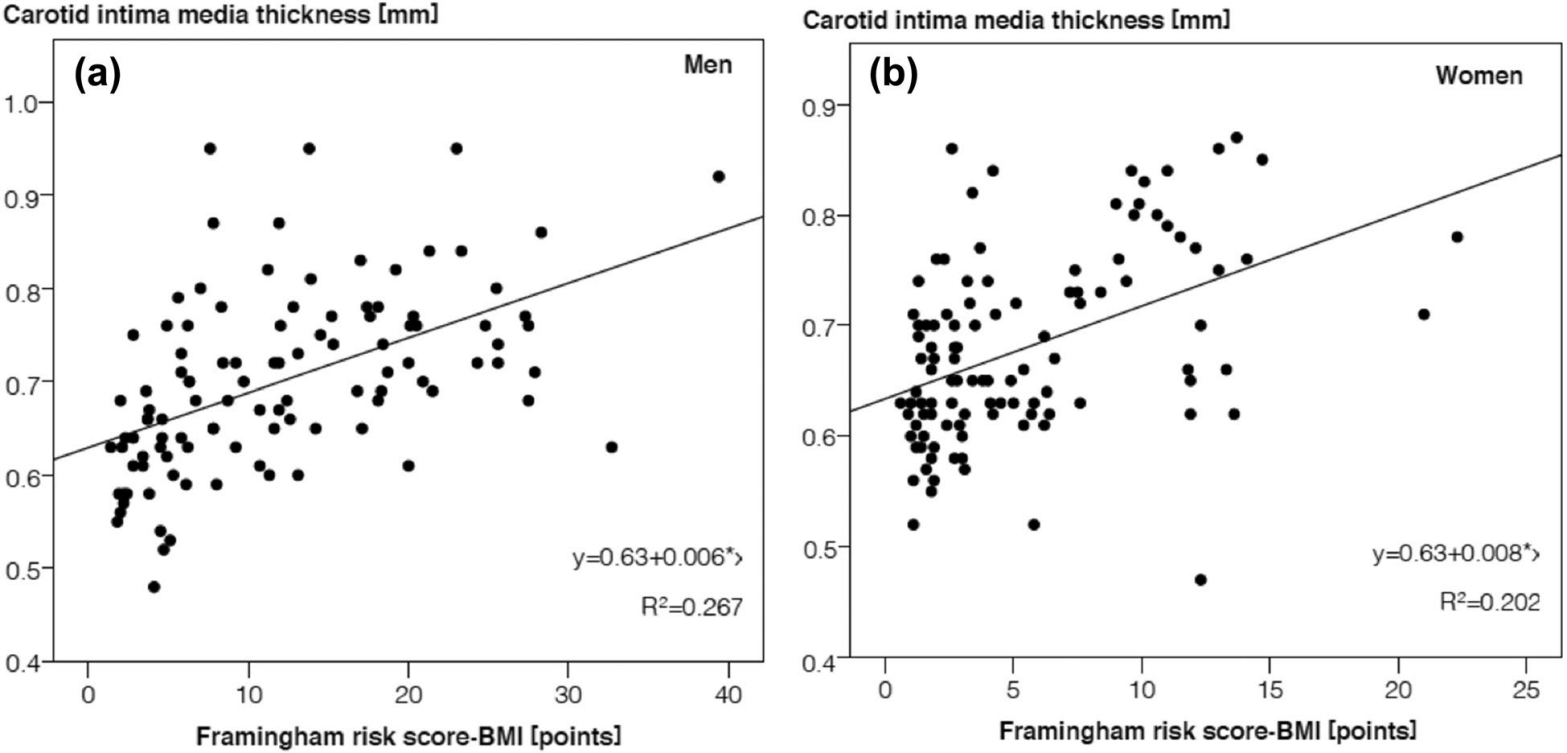
Univariate linear regression analysis of Framingham Risk Score-BMI and carotid intima–media thickness in men (**a**) and women (**b**)

In univariate linear regression analyses positive association between calculated scores and CIMT were observed for all risk scores. Again, the associations were stronger for men than for women and FRS-BMI, which showed numerically the strongest of the quite similar association with CIMT, predicted 26.7% of the CIMT variance in men and 20.2% in women (Fig. 1). Assuming a close linear relationship each FRS-BMI score point translated into 0.006 mm CIMT in men and 0.008 mm CIMT in women.

## Discussion

The present analysis of the STRATEGY study demonstrates a correlation of CIMT with values of common risk factors within the range of normal and with deduced risk prediction scores even in individuals at low cardiovascular risk lacking overt risk factors. Yet, the correlations were modest. Thus, both, clinical risk factors and CIMT may separately reflect a contribution to atherogenesis. Since prediction of cardio- and cerebrovascular events has been shown likewise for risk factors or risk scores and for the CIMT, based on the results of this study of only partial correlation of risk scores and CIMT it appears reasonable to assume an additive predictive power for the combination of the two approaches in individuals at low cardiovascular risk according to their risk factor profile. The perception of CIMT as an at least in part independent cardiovascular risk factor rather than merely a reflection of the common risk factors is in line with findings in the general population including subjects at clearly increased cardiovascular risk. However, at that stage of overt risk CIMT appears to add little to the risk predicted solely by risk factors [18].

The data of Table 2 illustrate that the cardiovascular risk factors of the studied individuals were on average in the range of normal despite clear gender differences. However, considering the variance, in individual participants some risk factors reached elevated levels though not in the range supposed to require therapy, e.g., the systolic blood pressure reached on average already the range of prehypertension. Thus, almost 50% of men were prehypertensive which bears a substantial cardiovascular risk. Also, a considerable number of men and women had a fasting glucose above the limit of prediabetes, which corresponds to a markedly elevated cardiovascular risk as well. In addition, the standard deviation of the LDL-cholesterol indicates a prevalence of individually clearly elevated cholesterol levels. This may in part explain the somewhat elevated average CIMT in the studied population, considering that in male and female infants the CIMT measures 0.3–0.5 mm [15–17].

In line with the presumption that CIMT may increase due to the conventional cardiovascular risk factors, we found highly significant correlations of the basic risk factors systolic blood pressure, fasting glucose and cholesterol with CIMT. Though age reached the highest correlation, it may just or in part be a surrogate of increasing number and severity of risk factors with age. Similarly, weight may merely express the prevalence of components of the metabolic syndrome. In accord, waist circumference as a measure of central obesity reached a higher and significant correlation than weight and BMI. Multivariate analysis yielded a strong significant correlation for age, while among the modifiable risk factors only systolic blood pressure reached significance in line with previous investigations [19, 20]. Similar results were reported for the correlation of age, gender and systolic blood pressure with CIMT [21].

However, since age may confound the effects of the individual risk factors due to their increase with age, a multivariate analysis was performed omitting age. The correlation of blood pressure, LDL- and HDL-cholesterol and fasting glucose with CIMT increased, which was substantial regarding blood pressure and fasting glucose, the latter of which reached significance in addition to blood pressure. In total, this analysis indicates that the sum of the modifiable clinical risk factors has to be taken into account when comparing their effect on cardiovascular risk with that of CIMT.

The individual point estimates of the calculated scores showed a linear relationship with CIMT. However, the coefficients of determination were rather low indicting a vast variation. In other words, more aspects than the considered parameters of conventional risk factors are responsible for the increase of CIMT. Thus, in individuals without overt cardiovascular risk factors CIMT may well add to the information of the risk scores with regard to the prediction of cardiovascular risk, which needs to be shown in large longitudinal studies, though.

The present findings of the STRATEGY study have several limitations that need to be addressed. First, the analysis is confined to primarily quite healthy volunteers characterized by a healthy lifestyle and a low cardiovascular risk profile, while individuals with overt and treated cardiovascular risk factors were excluded. However, this low-risk study cohort has been deliberately chosen as it represents the target population for which risk prediction is important to individualize and initiate necessary measures to prevent cardio- and cerebrovascular events at an early stage.

Second, the sample size of the study is limited, thus, analysis of a larger population may have allowed to detect significant associations between risk factors and CIMT and their differences with regard to gender. Also, determination of CIMT has followed the protocol of the SHIP-study for matters of conformity and to allow comparison. Automated edge detection may provide more precise results.

Third, some risk factors are underrepresented, e.g., smoking, and thus may be underscored. However, smoking is an obvious risk factor and needs to be stopped in any event. This also applies to markedly elevated lipids and plasma glucose in the range of diabetes. Thus, the findings and derived conclusions may not apply to populations at clearly increased vascular risk.

Fourth, in this context it may be borne in mind that there are more parameters that may be worth considering as to cardio- and cerebrovascular risk as further laboratory values like lipoprotein(a), lifestyle factors, sociodemographic determinants and psychological characteristics, which may add significant predictive power. Although plaque thickness may be considered, plaques are rare in individuals at low cardiovascular risk as in this study [22, 23].

In summary, despite these limitations, our data provide relevant information about two screening methods that share some predictive power, but also suggests that a combination of both may add predictive value in individuals at currently low vascular risk according to conventional risk scores. Such approach certainly requires further confirmation in a prospective cohort not only to prove an additional prognostic value, but also benefit as to the prevention of vascular events.

## Data Availability

The datasets used and analyzed during the current study are available from the corresponding author on reasonable request.

## Abbreviations

CIMT: Carotid intima–media thickness
LDL-cholesterol: Low-density lipoprotein-cholesterol
HDL-cholesterol: High-density lipoprotein
